# Questions about Using the Induced Membrane Technique to Manage Cases of Congenital Tibial Pseudarthrosis

**DOI:** 10.3390/cells12141918

**Published:** 2023-07-24

**Authors:** Céline Klein, Florelle Gindraux, Alain-Charles Masquelet, Romuald Mentaverri, Richard Gouron

**Affiliations:** 1 Department of Paediatric Orthopaedic Surgery, Amiens University Hospital, Jules Verne University of Picardie, F-80054 Amiens, France; gouron.richard@chu-amiens.fr; 2 MP3CV-EA7517, CURS–Amiens University Medical Center, Jules Verne University of Picardie, F-80025 Amiens, France; mentaverri.romuald@chu-amiens.fr; 3 Service D’orthopédie et Traumatologie Pédiatrique, CHU Amiens-Picardie, F-80054 Amiens CEDEX 1, France; 4 CHU Besançon, Service de Chirurgie Orthopédique, Traumatologique et Plastique, F-25000 Besançon, France; fgindraux@chu-besancon.fr; 5 Laboratoire de Nanomédecine, Imagerie, Université de Franche-Comté, Thérapeutique EA 4662 (LNIT), F-25000 Besançon, France; 6 Hôpital Saint Antoine, Sorbonne Université, F-75006 Paris, France; acmasquelet@free.fr; 7 Department of Biochemistry and Endocrine Biology, Amiens University Medical Center, Jules Verne University of Picardie, F-80025 Amiens, France

**Keywords:** induced membrane, congenital pseudarthrosis of the tibia, neurofibromatosis 1, Masquelet’s technique, leg, children

## Abstract

The induced membrane technique is an innovative approach for repairing critical bone defects and has been applied recently in patients with congenital pseudarthrosis of the tibia (CPT). CPT is frequently associated with neurofibromatosis type 1 (NF1). Here, we briefly describe the clinical results of the induced membrane technique in NF1-deficient patients with CPT and in an animal model of CPT. Furthermore, we discuss the hypotheses used to explain inconsistent outcomes for the induced membrane technique in CPT–especially when associated with NF1.

## 1. Introduction

Following on from our Special Issue on “Mineralized Tissues Repair and Regeneration 2.0”, we attempted to highlight issues in repairing defects for congenital pseudoarthrosis of the tibia (CPT) during neurofibromatosis type 1 (NF1).

CPT is a rare deformity, with an incidence of 1 per 140,000 births [1]. CPT results in anterior convex tibial bowing that worsens with growth. The bone tissue at the junction between the proximal and distal fragments of the tibia is extremely dystrophic, weak, and prone to fracture. CPT is most common in the distal tibia or at the junction of the middle and distal thirds of the tibia.

Several classifications have been described. The most commonly used include Crawford’s descriptive classification and Boyd’s prognostic classification [1,2]. The exact etiology of CPT is not known. Histological analysis of CPT reported the presence of fibrous hamartoma tissue with fibroblasts and a thickening periosteum surrounding the pseudoarthrosis [3,4,5]. The fibrous hamartoma tissue was described by Cho et al. as the key component of the CPT and was composed of cells with a mesenchymal stem lineage [3]. A hyalin cartilage, fibrocartilage, and features of enchondral ossification have also been described [6,7]. Fibrovascular tissue with the active, osteoclastic resorption of the cortex has been reported in dysplastic CPT [7]. The osteolytic components were observed more frequently in young children than skeletally mature patients [2]. In periosteum, the preferential differentiation of fibroblasts/myofibroblasts and multipotent mesenchymal cells leads to abnormalities in bone callus formation [8]. The periosteum might also be involved in the pathogenesis of CPT.

NF1 is present in 40–80% of cases of CPT [1]. In contrast, only 1–4% of children with NF1 have CPT [1]. The physiopathological mechanisms of bone and periosteal formation in CPT, in general, and NF1-associated CPT, in particular, are not fully understood.

The loss of function in the neurofibromin (the mutated protein in NF1) induces impairments in the Ras-mitogen-activated protein kinase (MAPK) pathway, which, in turn, impairs the differentiation of osteoblasts and osteoblastic progenitors. The increase in osteoclast activity caused by Ras overexpression might explain the physiopathology of CPT in general and the bone resorption and high fracture rate in particular [3]. In some cases of NF1-associated CPT, cells in the diseased tissue carry a doubly inactivated NF1 (due to somatic mutation of the *NF1* gene) [9,10,11]. However, Ippolito et al. failed to find any histological differences in the pseudarthrosis zone between the forms associated with NF1 and those that are not [7].

The surgical reconstruction of bone defects in CPT is still a challenge for the orthopedic surgeon, and there is disagreement regarding the best approach. Bone consolidation, the correction of the tibial axis, and functional results are not always optimal. Several surgical techniques can reportedly be used to achieve bone union and axis correction. Conventional treatments consist of the total resection of the pathological tissue and the correction of the tibia axis: the Ilizarov technique, vascularized bone transfers, and intramedullary nailing with a bone graft.

The induced membrane (IM) technique (also known as Masquelet’s technique) is an innovative, two-step approach for bone reconstruction and osteosynthesis between the proximal and distal bone segments [12]. Firstly, a poly(methylmethacrylate) spacer is placed in the bone defect, inducing the growth of an encapsulating membrane. In the second step, the spacer is removed, and the defect surrounding the IM is filled with cancellous bone graft [12]. The success of the IM technique is due to the membrane’s properties [13,14,15]. The use of the IM technique in cases of CPT has attracted interest; it allows the replacement of diseased bone tissue and periosteum by a rich vascularized membrane with significant osteo-inductive properties, a high growth factor content, and properties similar to those of periosteum [4,16,17]. In light of this, research has been conducted first in the clinic before being optimized in animal models. 

## 2. Clinical Observations

Pannier et al. achieved bone healing over four months in four out of five cases of CPT treated with the IM technique. The researchers reported nail migration, two non-unions, and a requirement for rods. Two patients required an inter tibiofibular graft for non-union, and one of the two had an extensive, dystrophic form of CPT [18]. Vigouroux et al. described ten cases of CPT treated with the IM technique (including the five patients in Pannier et al.’s study) and found that the mean time to consolidation was 23.1 months, with three re-fractures, and the need for an average of 3.4 interventions to achieve bone consolidation [19]. In Vigouroux et al.’s series, three patients experienced intramedullary nail complications with tibial nail migration and fibula nail exteriorization. At the last follow-up, one patient required tibial amputation. Dohin et al. studied three patients with CPT treated with IM and recombinant human bone morphogenetic protein-2 (rh-BMP2); the mean consolidation time was 18 months [20]. The first patient required several revisions: firstly, for distal non-union (treated with an additional bone graft, rh-BMP2, and modified osteosynthesis using a plate) and, secondly, for further treatment with rh-BMP-2 and a new plate for bone graft resorption. This case was complicated by a deep tissue infection, and the patient eventually had to undergo a below-knee amputation [20]. The second patient achieved bone consolidation at 28 weeks but presented a tibial shaft fracture two years later. The third patient required several surgical revisions for proximal non-union, fractures, and renewed osteosynthesis. Bone consolidation was eventually achieved in the latter two patients. Meselhy et al. reported a mean consolidation time of 25.3 weeks for 19 patients with CPT (mean age: 6.3 years) who were treated with the IM technique after the failure of other treatments [21]. The IM technique was combined with an autologous, free, non-vascularized fibular graft, an autologous iliac graft, and osteosynthesis using an intramedullary k-wire and an Ilizarov external fixator [21]. One patient experienced a tibial shaft fracture 3 months after surgery [21].

Our group used the IM technique to treat five patients with CPTs and NF1. In two patients, bone consolidation was achieved after 30 and 32 months, respectively [22]. One child had a junctional non-union at 56 months and had to wear a splint. A second child experienced the complete lysis of the graft and nail migration at 21 months; a new, two-step IM procedure was required for bone consolidation. A third child showed junctional non-union both proximally and distally at 18 months, with nail migration. In all cases, we used flexible intramedullary nails.

## 3. Animal Models

Several murine conditional *Nf1* knock-out models have been developed [10,23,24,25]. Although these models do not have exactly the same phenotype as in human CPT, they enable researchers to study the underlying mechanisms of NF1-associated CPT in the context of tibial fracture. El-Hoss et al. described a mouse model with local *Nf1* knock-out in healing fractures that used a Cre-expressing adenovirus [23]. The non-union rate in mutant mice was 50% higher than in wild-type mice [23]. Moreover, a fibrous non-union containing large TRAP+-like cells was observed in a midshaft fracture in double-knock-out *Nf1* mice [23]. Histological analysis of the *Nf1^null^* mouse fractures showed that the fracture site had been invaded by fibrous tissue and osteoclastic cells. These findings suggested that local *Nf1* knock-out in the fracture callus had the same histological characteristics as human CPT and impaired healing. In a study of wild-type and *Nf1^+/−^* mice, Schindeler et al. used mediodiaphyseal and lower 1/3 tibial fractures to mimic CPT [24]. When the osteotomy was performed in the mediodiaphyseal area, there was no difference between the two groups of mice. By contrast, when the osteotomy was performed on the lower third of the tibia, only 36% of the fractures in the N*f1*^+/−^ group healed fully (vs. 83% in the wild-type group). A histological analysis of the bone bridges in the N*f1^+/−^* group showed the presence of osteoclasts, cartilage, and fibrous tissue–confirming a previous study by Yang et al. in which N*f1^+/−^* mice had a greater intrinsic capacity for osteoclastogenesis than wild-type mice [26]. Based on the hypothesis of insufficient bone formation and excessive bone resorption, Schindeler et al. evaluated the synergic action of rh-BMP-2 and bisphosphonates in a murine *Nf1* knock-out mouse model, with encouraging results [24,27]. However, the outcomes of using BMP in the IM technique on human CPT vary from one study to another, and the administration of BMPs in children is subject to debate [28,29].

## 4. Discussion

Data from the literature and our preliminary results on the treatment of NF1-associated CPT with the IM technique highlight delays in consolidation, refractures, and graft resorptions–suggesting that the outcome is influenced by other unidentified factors and also by the intrinsic properties of the IM when formed in the context of neurofibromin’s loss of function [18]. In children without *NF1* mutations, bone healing after using the IM technique appears to be achieved quickly, with few complications or repeat operations [28,30,31,32,33,34,35].

The loss of a functional *NF1 gene* induced a neurofibromin loss of function and aberrant Ras-MAPK signaling. The intrinsic bone pathology with tibial pseudarthrosis (spontaneous fracture, failure to achieve bone union, etc.) might be explained by greater osteoclastic activity and more osteoclastic precursors due to impairments in the Ras-MAPK pathway [3,36].

Animal models offer perspectives that optimize critical defect bone fixation and IM formation. It has been shown that the IMs in humans and animals are similar with regard to the matrix composition and the cell content [13]. Moreover, as we have shown previously, the new mouse knock-out models accurately mimic the pathophysiology of CPT.

During the formation of the IM, functional proteins involved in osteoblast differentiation and proliferation (such as BMP-2, transforming growth factor beta, vascular endothelial growth factor A, and von Willebrand factor) were expressed [13,37]. Given that neurofibromin is involved in the MAPK-Smad signaling pathway, Tang et al. assessed the osteogenic differentiation of mesenchymal stem cells when exposed to protein extracts from rat IM and analyzed the expression of osteogenic markers (Runx2, collagen I, osteocalcin, and alkaline phosphatase) using RT-qPCR assays and Western blots [38]. By applying specific inhibitors of Smad1/5/8 (LDN-193189) and ERK1/2 (U0126), Tang et al. found that IMs exhibited osteogenic properties in relation to the Smad1/5/8 and MAPK pathway activation within rat mesenchymal stem cells. The combined treatment of IM with LDN-193189 and U0126 significantly inhibited an increase in Runx2, collagen I, and osteoclacin expression induced by IM proteins. These results suggest that proteins in IM promote the osteogenic differentiation of mesenchymal stem cells [38].

In view of these data, we hypothesized that the Smad and MAPK pathways within the IM were modified by neurofibromin’s loss of function and the presence of impairments in the Ras-MAPK pathway. This hypothesis could explain the inconsistent outcomes of IM in patients with NF1 patients, and is further strengthened by recent reports of somatic mono-allelic *NF1* inactivation in the pseudarthrosis periosteum in non-NF1-CPT [39]. Furthermore, it was shown recently that IM’s intrinsic properties influence clinical outcomes [14,15,40].

Another (more mechanical) hypothesis might also explain these inconsistent results. Indeed, we now know that the results of the IM technique are conditioned by the stability of osteosynthesis [41]. However, patients are generally operated on at a very young age, and flexible intramedullary nails do not provide very stable fixation.

## 5. Conclusions

In the surgical treatment of CPT (especially in NF1-deficient patients), it is difficult to obtain bone consolidation, axis tibia correction, and good functional results. The IM technique is a very interesting option; however, the outcomes are inconsistent for several reasons, including mechanical stability and the IM’s properties. The results of our translational research on CPT and IM (i.e., the combination of fundamental research and clinical studies) encourage us to continue our work in this direction.
